# A modular computational framework for medical digital twins

**DOI:** 10.1073/pnas.2024287118

**Published:** 2021-05-10

**Authors:** J. Masison, J. Beezley, Y. Mei, HAL Ribeiro, A. C. Knapp, L. Sordo Vieira, B. Adhikari, Y. Scindia, M. Grauer, B. Helba, W. Schroeder, B. Mehrad, R. Laubenbacher

**Affiliations:** ^a^Center for Quantitative Medicine, University of Connecticut Health Center, Farmington, CT 06032;; ^b^Kitware Inc., Clifton Park, NY 12065;; ^c^Department of Computer Science, University of Michigan, Ann Arbor, MI 48109;; ^d^Department of Medicine, University of Florida, Gainesville, FL 32611

**Keywords:** medical digital twin, modular design, multiscale computational model

## Abstract

This paper presents a modular software design for the construction of computational modeling technology that will help implement precision medicine. In analogy to a common industrial strategy used for preventive maintenance of engineered products, medical digital twins are computational models of disease processes calibrated to individual patients using multiple heterogeneous data streams. They have the potential to help improve diagnosis, prognosis, and personalized treatment for a wide range of medical conditions. Their large-scale development relies on both mechanistic and data-driven techniques and requires the integration and ongoing update of multiple component models developed across many different laboratories. Distributed model building and integration requires an open-source modular software platform for the integration and simulation of models that is scalable and supports a decentralized, community-based model building process. This paper presents such a platform, including a case study in an animal model of a respiratory fungal infection.

## Significance

The digital twin paradigm holds great promise for medicine, even though many technical and scientific challenges remain to be overcome, most importantly the efficient integration of many heterogeneous component models. This is an unsolved problem even in industry. It has long been understood that such models need to be built in a modular fashion, connecting together component models of individual biological processes. In conventional implementations, however, the dependency structure of the modules reflects the dependencies among these processes, making it all but impossible to modify or expand the digital twin without breaking it. This paper presents a fundamental reorganization of the internal digital twin architecture into a hub-and-spokes design that completely circumvents this dependency problem.

Type I diabetics now have available a medical device, an “artificial pancreas.” It is based on a mathematical model of glucose metabolism calibrated to an individual patient. The model, running on a smartphone-like device, receives real-time blood glucose levels from a sensor in the patient, calculates required insulin needs, and drives a pump attached to the patient that injects the appropriate dose of insulin (1). The patient gains quality of life and is less likely to end up in an emergency room with an insulin overdose. The artificial pancreas is an example of a medical digital twin, in analogy to a common strategy in industry. As an example, airplane engines are designed using a complex mathematical model. This model is then calibrated to an individual engine using real-time performance data continuously streamed to the manufacturer, becoming that specific engine’s digital twin. The manufacturer, in consultation with the airline, can use the digital twin for purposes such as preventive maintenance recommendations. Human beings are far more complex than airplane engines, but the digital twin concept has a clear analogy in medicine despite our incomplete understanding of the determinants of health and disease. In cardiology, prediction using personalized computational models informs interventions (2). For other examples, see refs. 3–8.

Digital twins in biomedicine need to evolve continuously to represent the current state of knowledge and data. A large-scale implementation of the digital twin paradigm for human health requires the construction and execution of highly complex models composed of several component models which span multiple spatial and temporal scales. To realize the full potential of the digital twin concept, a flexible software development platform is needed that enables multidisciplinary and distributed teams to work together, supports reproducibility, and facilitates the integration of data and component models. Design patterns common to “traditional” model implementations impair the development of integrative digital twins. Some of these patterns include the following: 1) lack of transparency in the implementation of computational models, 2) intertwined component models and simulation processes dependent on each other, 3) use of incompatible data structures and computer languages, 4) brittle architectures that do not easily accommodate extensions of a model, and 5) software environments that do not easily support distributed collaboration. Solutions to these challenges are still largely lacking, not only in biomedicine (9).

To address these problems, we have developed an approach based on an open-source, highly modularized computational representation of a digital twin architecture. While the concept of modular design of models and software is well established, the way modules are assembled generally suffers from the shortcomings listed above. The central principle of the architecture we have developed is the separation of computational algorithms for the different dynamic processes, eliminating dependencies that make model modifications and extensions cumbersome or impossible in complex models. It also features the separation of computational algorithms from data, in the sense that all data describing the global model state, including model parameters, are separate from the individual computational modules in a “hub-and-spoke” transparent architecture optimally designed to facilitate extension and modification and web enabled for distributed collaboration. This approach differs fundamentally from the conventional approach to building and simulating such models in biomedicine, as described below.

We demonstrate this design approach and its advantages by applying it to the published model in ref. 10 of the early immune response to a respiratory infection by the fungus *Aspergillus fumigatus*, a dimorphic fungus that is ubiquitous and causes difficult-to-treat infections in immunocompromised patients, with high mortality. The emergence of strains resistant to first-line antifungal drugs makes the development of host-centric interventions a high priority. The agent-based model in ref. 10 could form the basis for a digital twin of some relevant functions of lung immunity used to simulate interventions personalized by data characterizing a patient’s immune status. We restructure this model using the modular design approach. Altogether, the technology we have developed makes it possible to design, calibrate, validate, and use multiscale computational models by a distributed team. Additionally, the principles developed are transferable to many other complex digital twin modeling scenarios.

## Results

**Fig. 1.**
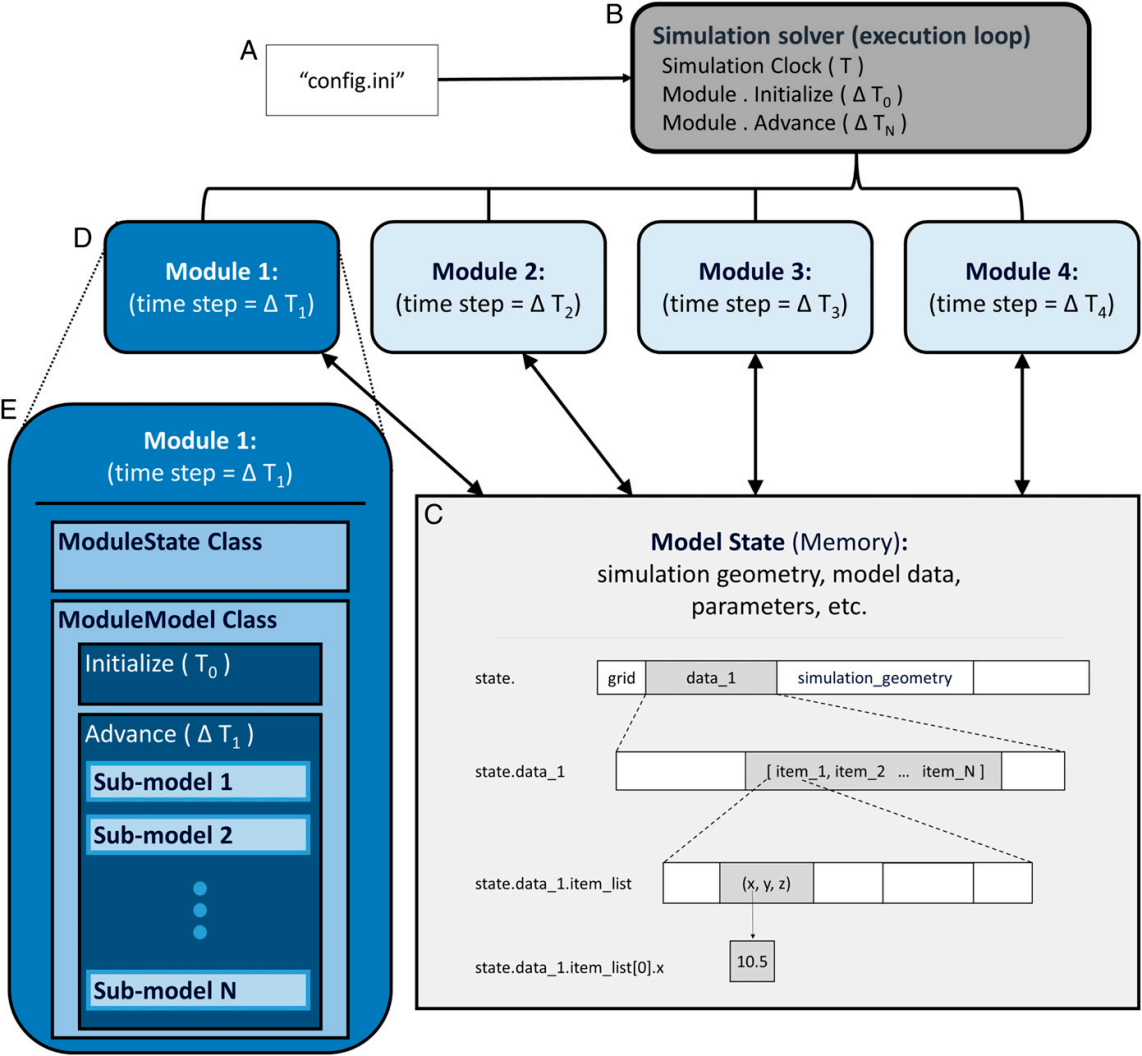
Modular model components. The modular design implementation contains four components. (*A*) The runtime configuration file that contains all configuration and parameter settings for a given simulation run (“config.ini”). (*B*) A simulation solver that reads the configuration (config.ini) and constructs, initializes, and advances the simulation in time by executing each module according to its inherent time scale. (*C*) The model state contains all data describing the state of the model at a given point in time, including any physical space geometry, and states of model objects. In this example, the model includes a spatial component. The model state is a contiguous block of memory as shown by the partitioned rectangle, with the hierarchical Python-referencing syntax shown to the left of the representation. (*D*) Each module consists of a computational model that takes all input data from the model state and stores none itself. (*E*) These modules extend classes provided as part of the simulation framework, “ModuleState” and “ModuleModel,” which handle the connection to the simulation solver and model state access, so the developer only needs to consider the biological additions to the model. Extending the ModuleState results in the fields defined in the extending class being appended to the model state. The “initialize” and “advance” functions in the ModuleModel extension will be called by the simulation solver, so the module can participate in the simulation.

The core principle underlying the highly modularized architecture we propose here is to treat each dynamic biological process in the model, or related collections of processes, as a separate module of the digital twin. In a biological context, a molecular module might contain the algorithms for diffusion and transport of that molecule, while a cellular one could contain models related to that cell’s function. The individual modules are only indirectly connected by communicating through a central data structure, the global model state, rather than passing data to each other directly. This prevents any direct dependencies between the computational portion of modules, a key feature that enables the model to be readily extended or modified. The global model state is the repository for all data describing the state of the simulated model at a given point in time, including any information about the underlying physical structure, if included, and variable states of all computational models in the modules. All computational algorithms, on the other hand, are contained in the modules, providing a clear separation between the model and the data on which it operates during model simulation. The resulting computational structure naturally separates model components so that they may be validated by the distinct modalities natural for each of the dynamic processes in the model, facilitating continued model refinement and personalization. Our implementation contains four components: 1) a runtime configuration file, 2) a global model state, 3) modules, and 4) a simulation framework that controls simulation runtime and provides data structures and algorithms useful for the development of modules. These four components and their relationship are represented in Fig. 1, providing a coherent and easily discernible structure for the model components and their dependencies.

### Model State.

The hub of the model structure is the shared global model state that integrates all runtime data describing the state of all components of the model and provides a snapshot in time of the model simulation. It is the central and only dataset used during a simulation run. At the same time, it does not contain any parts of the computational and mathematical instructions of the model. The model may contain a spatial component, such as a tissue or organ, represented either abstracted or in physiological detail. The model state can capture any spatial heterogeneity in its data structure through a grid or other spatial architecture. As a digital twin is modified or expanded by either changing or deleting a dynamic process captured by the model or adding a new one, the structure of the model state is modified accordingly by changing or deleting existing data fields or adding new data fields corresponding to the added biology. The result is a clear operational separation between the representation of the physical system of interest and the computational instantiation of the dynamic processes that drive its temporal evolution and serves as the basis of any operational predictions. At the same time, the model state is partitioned in a way that allows individual modules access to the part of the data that is used as input for their component model (see below). The technical details can be found within the *Methods*.

### Modules.

**Fig. 2.**
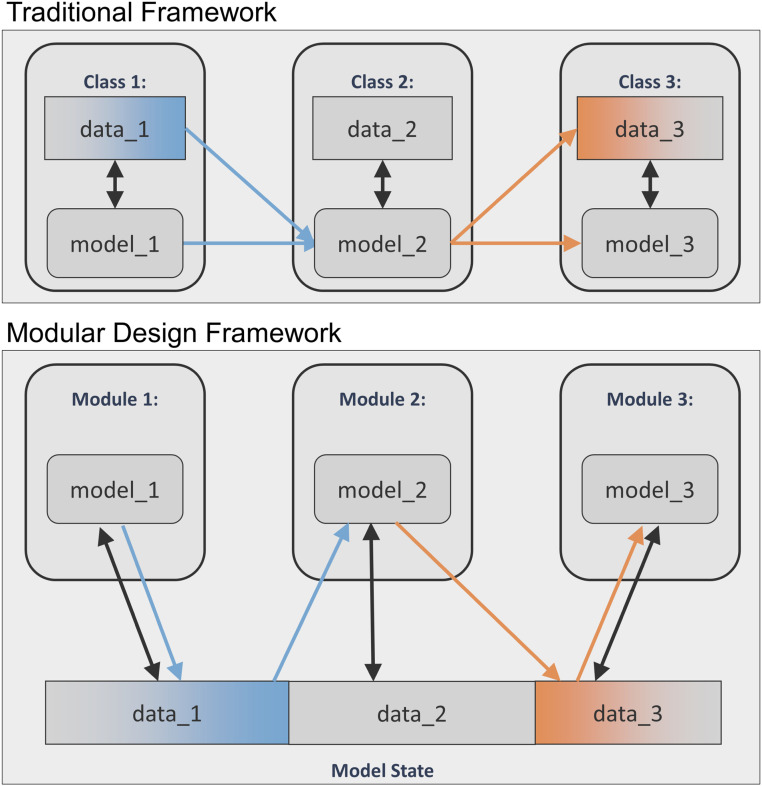
Modular design framework enhancements over conventional model structure. In this simplified scenario, a model consists of three generic processes with dependencies. In both implementations, processes are separated into three distinct representations, modeled by functions (model_1, model_2, and model_3) that update data (data_1, data_2, and data_3) at each time step, with interactions indicated by arrows. In the above example, process 1 is intertwined with process 2 and process 2 with process 3. While the traditional architecture (*Top*) results in Class_2 depending on Class_1 (via model_2 depending on the Class 1 model and data API), the modular architecture (*Bottom*) results in Module_2 relying only on a subset of the global data defined by Module_1’s ModuleState (blue data_1). In contrast to the failure to run if model_1 changes in the traditional case, Module_1’s submodel (model_1) can change independently of the ModuleState (data_1) and preserve the validity of the Module_2 model (model_2). In the general situation, in which there is a new state that needs to be added to the module’s ModuleState (1) to support such submodel changes, this will likewise leave the other module (2) unaffected. If there are parts of the ModuleState (1) to be removed, this is the one case that could require alteration of other modules (2). However, in this case, regardless of the design, removal of a biological interaction demands that both classes involved be altered. Likewise, if new biology is added affecting multiple classes, both designs require changes to their models and inputs and outputs.

As explained, the global model state consists of a data structure that captures the biology incorporated in the model and the data characterizing the model state at a given time during a simulation run. The entire computational infrastructure is contained in a collection of modules, one for each dynamic process incorporated in the model. Each module consists of a computational model that takes as input certain data from the model state used as model input. No data are stored in the modules themselves. The computational model in the module is simulated by reading data from the model state, carrying out a model iteration using this data to obtain a new set of variable states and updating the model state with the new set of data. This computational model can be a continuous model such as an ordinary differential equation, a discrete model such as an agent-based model or Boolean network, or a hybrid of continuous and discrete components. This sort of hybridization is accomplished by discretizing space and advancing the continuous parts of models over time intervals that are punctuated by ticks of the discrete parts. The read/write relationship connecting the module to the model state is the only connection of a module to the model. In particular, there is no direct interaction between the different modules. The resulting hub-and-spoke model/software structure is transparent and avoids the type of cross-module dependencies that would otherwise make module alteration or addition difficult, as is frequently the case for complex models in the conventional architecture as shown in Fig. 2. Module dependencies on particular libraries are explicit as part of the package installation, contributing to reproducibility of model simulations and predictions. Another advantage of the modular structure is the clear separation of the different data types that feed into the overall model, either one time, periodic, or streaming, as individual sensors or experiments typically correspond to individual dynamic processes.

In order to provide a common interface for modules to interact with the model state, each module is expected to provide a “ModuleState” subclass that defines the data structures relevant to that module to be stored within the model state. This subclass object resembles a standard Python “dataclass” with additional validation and serialization methods. The *Discussion* section below includes a discussion of interfaces with other programming languages. Simple scalar values such as integer or floating-point numbers, Boolean values, strings, and NumPy arrays (11) of the same can be added directly to this object handled by the superclass implementation. The ModuleState object plays the largest role in eliminating the extensibility issues that plague conventional implementations. It does this by preventing function calls as a means of interaction between component models and between models and data. Instead of utilizing another class’s functional application programming interface (API), each module presents a pure data API in which each module’s model interacts only indirectly with other modules through reading and writing to the global model state. The key consequence of this structure is that changes in the computational algorithms included in a given module have no effect on the algorithms of other modules, only on their parameters and data. This makes the entire architecture robust to algorithmic modifications and to expansion of the model by other modules and features. In a conventional implementation, such modifications require extensive effort to account for downstream effects in the program structure.

### Model Simulation.

The fundamental operation to be carried out with a model is to specify an initial configuration of the model state and apply the computational algorithms in the modules in discrete time steps to simulate the evolution of the model state from this initial state. This operation is carried out by the simulation solver, one of the components of the simulation framework. The solver contains an execution loop that advances the simulation in time by executing each module according to its inherent time scale. The simulation is divided into three stages: construction, initialization, and time stepping. During construction, the runtime configuration file, a file with general simulation configuration information (simulation length, global time step, modules to include, etc.) and all parameters for the models inside each module for a given simulation run, is read and all necessary memory is allocated within the model state. During the second stage, each module accesses the model state and reads all parameters specific to its setup, the part of the model state needed to initialize its computational algorithms, and any other needed setup information provided by the runtime configuration file.

During the third and final stage, the simulation begins looping through time iterations, passing parts of the model state through each module one at a time. At this point, a choice needs to be made about the order in which the modules are executed and updated. This is a special instance of a more general question about the simulation of models built from components, and there have been several theoretical studies of the impact of different update orders on model dynamics by us and others; see refs. 12 and 13. The solution typically employed is to choose a particular order. See *Methods* for details of our solution to this problem.

### Comparison between Modular and Conventional Model Architecture.

As described above, the key difference between conventional model architectures and the hub-and-spoke modular design we propose is that it eliminates direct dependencies between different model components, enabling modification of model components without having to trace the downstream consequences of changes in the model logic in one component into other components. Fig. 2 illustrates this difference for the case of three generic dynamic processes that depend on each other.

Lastly, we address the issue of data fields that are common to several modules, a potential source of problems in conventional model architectures. Suppose two or more modules have the same data field as input or output. For instance, two types of immune cells, *A*1 and *A*2, may synthesize and secrete the same cytokine (*C*). In addition, another module *A*3 might modify *C* through a partial differential equation (PDE) model that simulates the diffusion of *C* in space. The global model state contains a unique data field for *C* that is read and/or modified by all three modules. No duplication of data occurs, even as further modules are added that also utilize *C*. If one of the modules is removed, then only its effect on *C* is removed rather than *C* itself. Technically, this removal is accomplished by disabling both the initialize and advance functions for the module being removed, thereby removing everything related to that module’s computational model but preserving the model state. Thus, there is no true ownership of data fields by modules in the model, thereby avoiding issues of data field duplication as well as loss of data fields through the removal of modules.

## A Case Study: The Immune Response to Respiratory Fungal Infections

In order to provide a concrete illustration of the advantages of this modular design approach, we apply it to the dynamic computational model in ref. 10. This model captures part of the innate immune response to the respiratory fungal pathogen *A. fumigatus*, the causative agent of invasive aspergillosis, one of the most common fungal infections in immunocompromised hosts, carrying a poor prognosis. The increasing use of immunosuppressive therapies in transplantation and cancer has dramatically increased suffering and death from this infection, and this trend is expected to continue. *Aspergillus* spores are ubiquitous in the environment. Immunocompetent hosts clear the inhaled spores without developing disease, but individuals with impaired immunity are susceptible to a life-threatening respiratory infection that can disseminate to other organs. The infection is typically initiated by a small number of inhaled fungal spores that attach to the lung epithelium and then follow a progression of swelling, germinating, and growing into hyphal structures that ultimately invade the interstitial space and vasculature. The innate immune response is triggered by signaling from resident alveolar macrophages and epithelial cells, which recruit various immune cells from the circulation, mainly neutrophils and monocyte-derived macrophages/dendritic cells.

Current therapeutic approaches have been focused primarily on the pathogen, but a better understanding of the components of the host defense may lead to the development of new treatments. In particular, restricting iron availability to the pathogen is a critical mechanism of antimicrobial host defense; conversely, successful pathogens have evolved potent mechanisms for scavenging iron from the host. This competition has the potential to be harnessed therapeutically. The motivation for the model in ref. 10 is the development of a simulation tool to explore the role of iron in invasive aspergillosis across biochemical and biophysical conditions in search of therapeutic mechanisms.

**Fig. 3.**
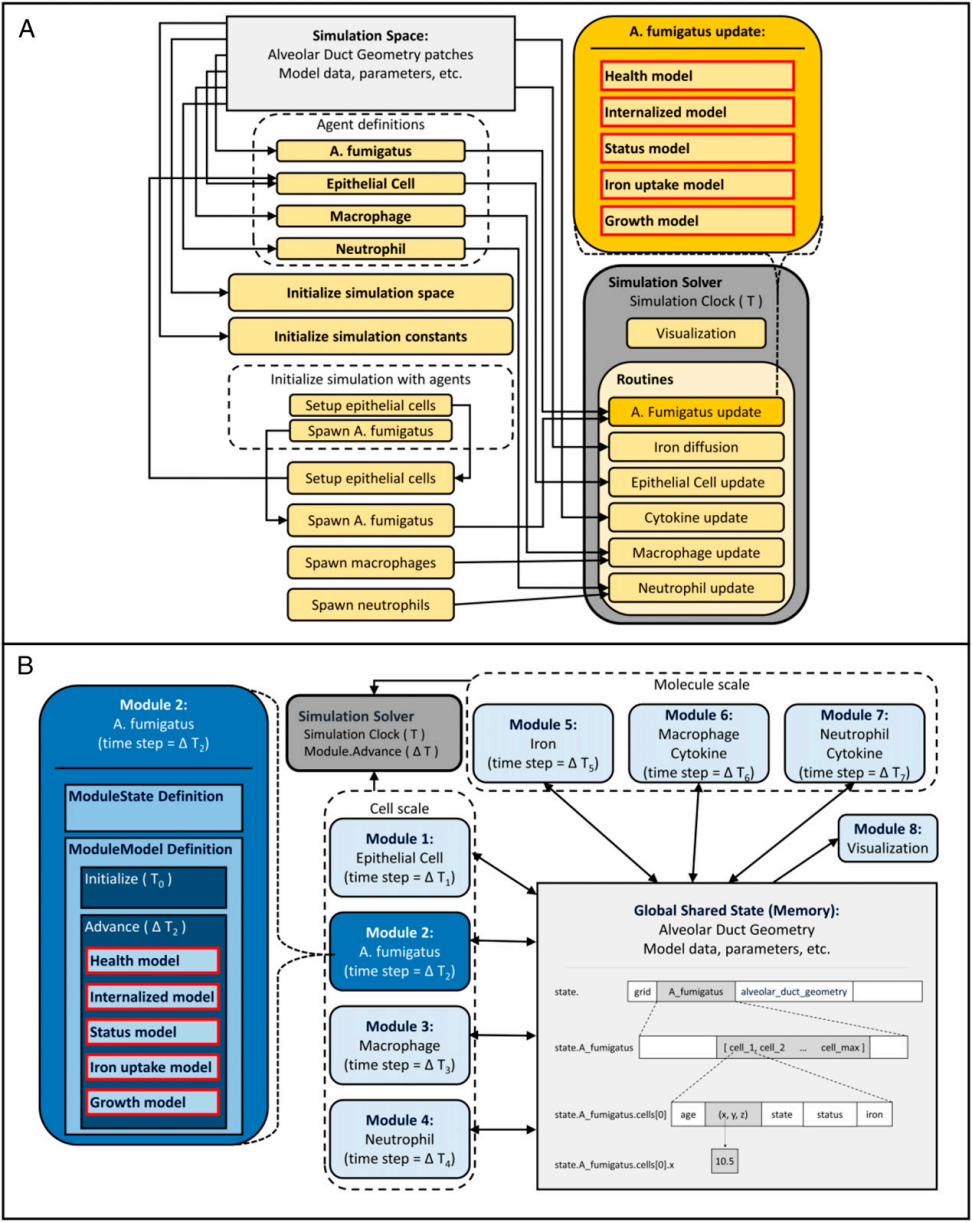
Comparison between modular and conventional model implementation. Dependencies between components derived from source code and written description in the absence of code are outlined. (*A*) NetLogo model. An object-oriented model with a high level of interconnectedness among components. (*B*) Modular design structure. A hub-and-spoke model in which modules (spokes) interact directly only with the shared global state (the hub).

This model can serve as an appropriate use/case for the modular framework we have described because it incorporates many of the features found in mechanistic models that might constitute or be part of a digital twin in biomedicine. Namely, it includes a multifaceted, multiscale response to a perturbation of a homeostatic physiological process, the hallmark of a wide range of medical conditions. As is often the case, an anatomical component, an alveolar duct in this case, is involved, providing a spatial component. Moreover, the model is composed of several component models, ranging from intracellular models to cell movement in lung tissue as well as diffusion of several molecules across the tissue. Below, we describe the model and compare its two implementations, captured in Fig. 3. We demonstrate that the modular implementation vastly simplifies its internal complexity. We also demonstrate the ease of adding new biological components to the model, arguably the most important advantage of this framework, as it facilitates the continued expansion and improvement of an existing model.

### High-Level Detail of the Model.

The model in ref. 10 is a so-called agent-based model, a widely used framework that is particularly suitable for modeling spatially heterogeneous processes. It also typically presents the biggest challenge to model reproducibility because of the lack of a set of underlying equations. Different types of “agents,” such as immune cells or fungal spores, navigate a spatial environment based on a set of rules that also govern the interaction of agents with each other, such as macrophages phagocytosing fungal spores. Movement and interactions evolve in discrete time across the three-dimensional voxelized space, representing a small portion of lung tissue in our case. Molecules such as cytokines diffuse across the tissue, also in time- and space-discrete steps. The model was implemented in the popular agent-based modeling platform NetLogo (18). Our implementation of the model is based on the article’s supplemental materials, including a description of the model in the so-called Overview, Design concepts, and Details protocol (14), a standardized way to describe agent-based models, which mitigates somewhat the lack of a well-developed markup language, such as that which exists for ODE models in systems biology markup language (15). However, important details and parameters are not included, which makes it impossible to reproduce model dynamics exactly. We note that most published models do not include such a description, making agent-based models notoriously difficult to reproduce (16).

### Separation of Dynamic Processes into Modules.

We illustrate the key modular design concepts using the internalization of fungal spores by epithelial cells in the lining of the alveolar duct. The structure of the two implementations of this process is shown in Fig. 3. It involves two agents that can occupy the same voxel, that is, are in close physical proximity, an epithelial cell and a fungal cell, and the encounter results in the modification of the states of both agents. Each agent’s state is determined by a computational model that takes as input all or some of the data from the model state that describe the voxel the agent is located in, including data about other agents present in the voxel. In this example, we will assume that an epithelial cell as well as a fungal cell are present. In the implementation in ref. 10, the two agents would exchange data through their respective APIs, creating a direct dependency between the two agents and, hence, a dependency between the two submodels representing the agents.

In contrast, in the modular design, the epithelial cell module accesses the global model state and extracts data about the voxel it resides in at that time. Using this data, such as presence of fungal cells, as input, the module’s model computes a probabilistic function determining the action of the epithelial cell. This action may include phagocytosis of the fungal spore, which is accomplished by both updating the epithelial cell’s data and by writing a flag for internalization to the spore’s portion of the global state. Thus, the fungal cell module and the epithelial cell module, each containing a computational algorithm, never interact directly but only indirectly through modifications of data in the model state accessed by both. We emphasize that in this way neither module depends on the functional API of the other one to carry out phagocytosis. If changes are made to the model in the fungal module, they have no effect on the epithelial module at the level of code. Furthermore, additions of parameters or data fields to a model, as a result of new biology, get simply added to the global state. By contrast, if the epithelial cell model included a function defined in part by the API of a fungal cell, such as in ref. 10, then, if that API changed as a result of new biology being incorporated into that module, every other module utilizing that API would potentially break during model execution.

### Comparison of Implementations.

Our “hub-and-spoke” modular architecture has several advantages over a conventional model implementation. In the traditional model, there is a high level of interconnectedness among components. The model is structured in an object-oriented way, in which objects model individual types of actors, each encapsulating its own data and providing functional API-mediating interactions between it and other parts of the model; see Fig. 3*A*. As an example, the *A. fumigatus* update routine is expanded to show the five models it contains, dependencies of which are shown by the arrows to the *A. fumigatus* update box. The result is that the simulation space, data, and parameters representing the model state at a particular time point as well as the model itself are all tied into object definitions and functions, with values hidden across agent definitions, the initializations, and the routines. Understanding of one part of the model requires a developer to read through most of the source code before attempting a minor change to a model because of the interconnectedness leading to brittleness at runtime.

In contrast, the data for the modular structure are contained entirely in a centralized data structure. We restructured the NetLogo model to dismantle the dependencies and conform to the modular principles. This involves converting routines as represented in the NetLogo model into modules, the most important step being a reconceptualization of the object-oriented structure to separate the dynamic process from the data it operates on. As a result, the implementation of the submodels contained within the routines changes, but crucially, the logic is preserved. This can be seen in Module 2’s replacement of the *A. fumigatus* update routine (Fig. 3*B*). The same five models are implemented but within the advance function in the modular framework instead of the update routine. The reduction in dependencies is displayed, as the arrows are only from modules to data in the model state. A developer needs only to understand the shared data in the model state, which are pertinent to a particular module, in order to understand how to make alterations to the module or to use it for the development of a new module.

**Fig. 4.**
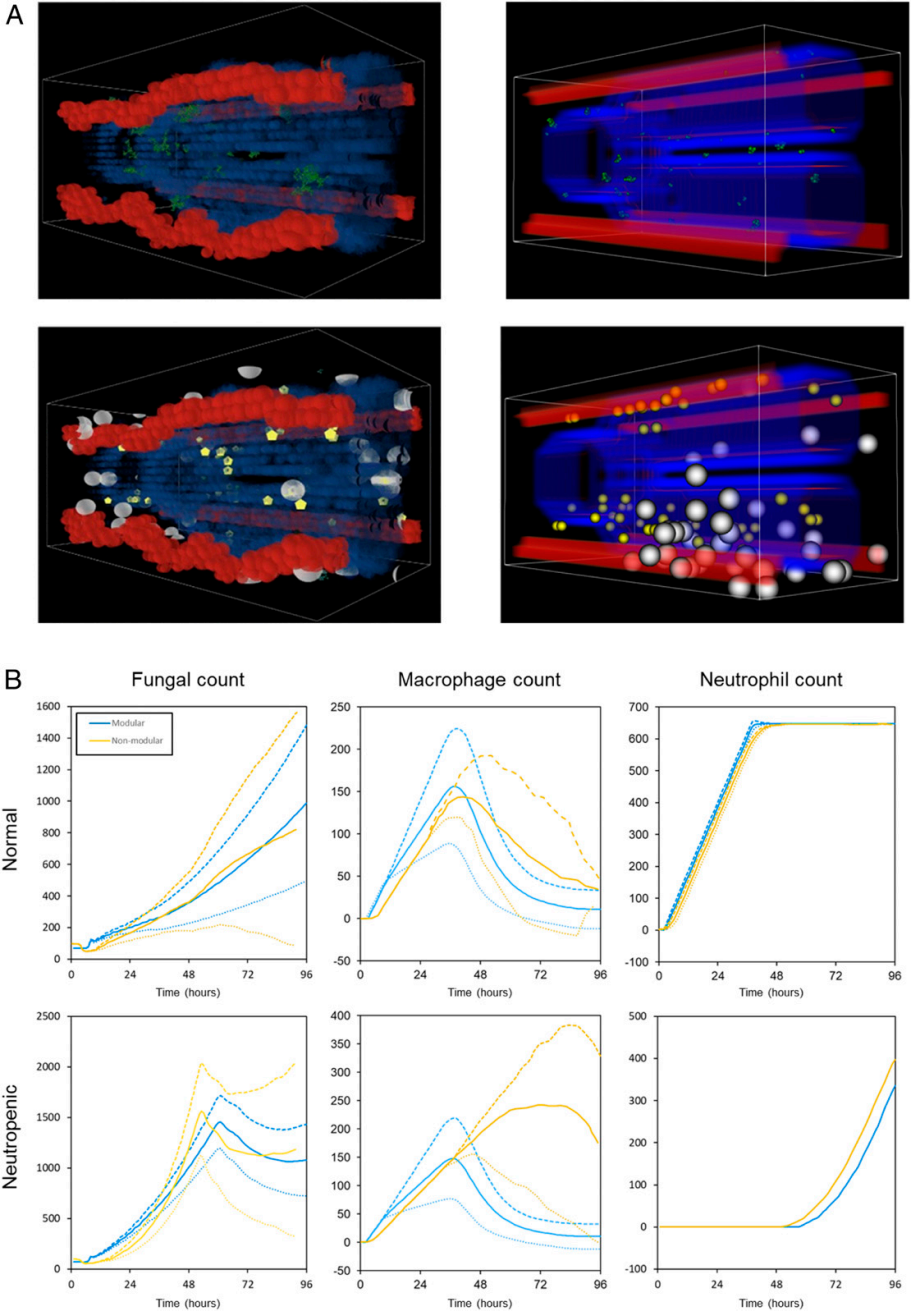
(*A*) Simulation space. Both implementations (nonmodular left and modular right) share a similar simulation space. Both have a pair of alveolar ducts lined by epithelium branching from a single-source duct (where fungal cells lodge and germinate; green spheres in top row) and four blood vessels that run across the space parallel to the duct. The blood vessels are the source of recruited macrophages (white spheres), and neutrophils (yellow circles) are recruited (bottom row). The remaining space is filled with nonspecific interstitial tissue. (*B*) Simulation results. The panels show the results of both the modular (blue) and nonmodular (yellow) simulations for fungal cell count, macrophage cell count, and neutrophil cell count in both the normal immune response (*Top*) and neutropenic immune response (*Bottom*). Since both models are stochastic, they were simulated multiple times, beginning with the same initialization, and we have plotted averages and SDs. In the panels, the solid line is the mean, the long dashed line is one SD above the mean, and the short dashed line is one SD below the mean for 200 simulations. The figures were created using the code and configuration from release “version 0.1.1.”

The modular reimplementation recreates the physical space over which model dynamics unfold, including a visualization tool (see also the *Discussion*). The fact that the description of the model in ref. 10 is incomplete, in addition to the fact that the model is stochastic, makes it challenging to replicate model dynamics exactly. We show in Fig. 4 the corresponding visualizations of the model as well as the time course evolution of some of the key model features, such as cell counts and concentrations of different molecules. The models agree strongly, such that complete information and correspondence to actual code is available. The neutrophil count serves as an example of this feature. It is most closely reproduced (Fig. 4*B*, column 3) because the neutrophil component of the model was documented fully. There was a code reference that matched the paper description exactly, and all the parameters were available in a single location, all on the same scale. Differences, such as macrophage counts or iron concentration over time, on the other hand, are due to ambiguities and the lack of information in the model description.

## Modular Design Facilitates Model Expansion

The modular architecture is specifically designed to make the process of model expansion and alteration not just feasible but quite straightforward. We illustrate this simplicity by adding a module to the modular implementation of the model in ref. 10 that introduces the effect of hepcidin, a hormone synthesized and secreted mainly by the liver. The innate immune response uses hepcidin to limit the pathogen’s access to iron by inhibiting its export from the intracellular space.

**Fig. 5.**
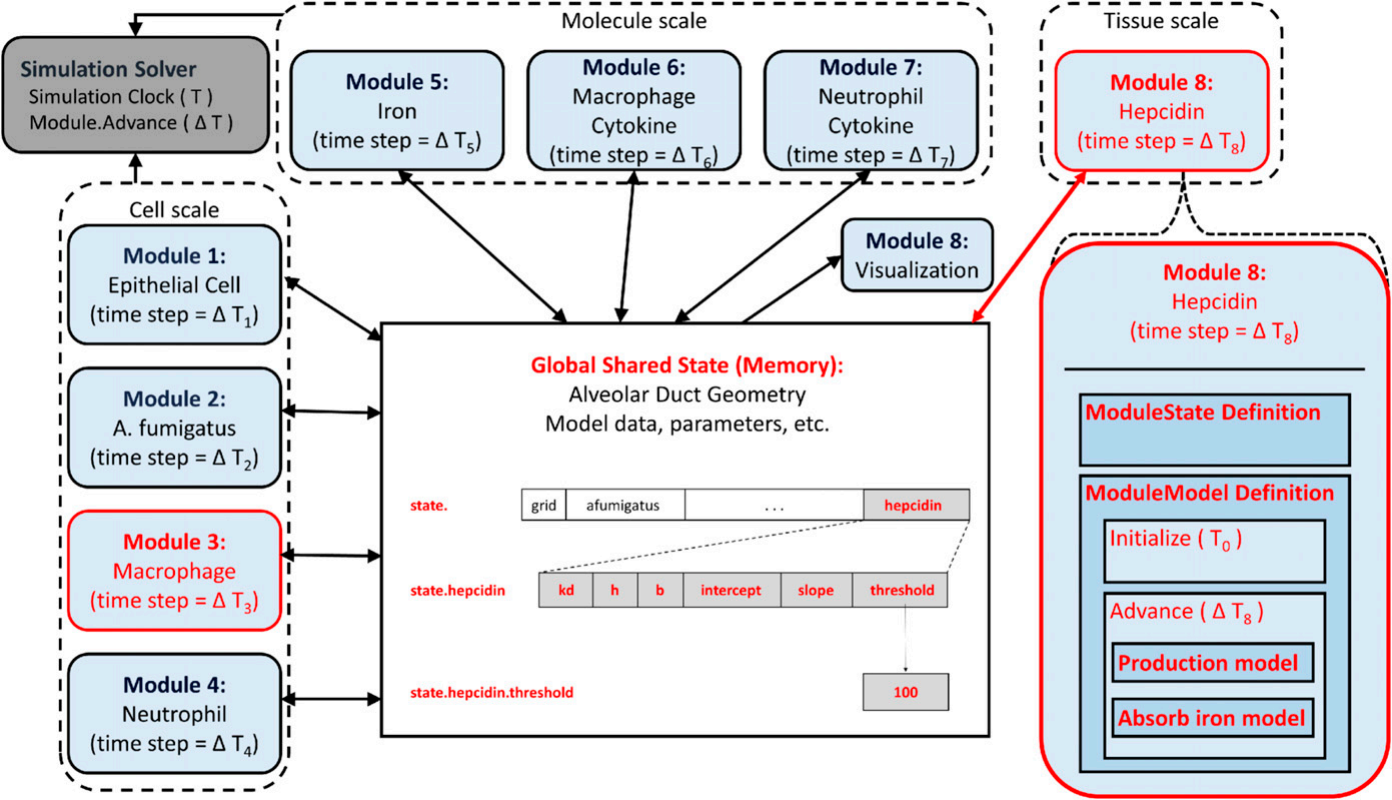
Adding a module to the model. To add the process of hepcidin regulation of macrophage iron handling to the existing model, we only need to create or change the components highlighted in red; a new module (Module 8: Hepcidin) is added with a ModuleState and ModuleModel definition, a new section in the configuration file, and any changes to the existing module’s models (Macrophage) required by the incorporation of a new biological factor. Using the ModuleState and ModuleModel superclasses makes this process of addition very simple, as the only new code is related to the new hepcidin biology, with the connection to the rest of the simulation handled by the components of the modular framework. The new code will exist in three places: 1) in a listing of the new model state under the extension of the ModuleState, 2) in the implementation of the “Production model” and “Absorb iron model” within the advance function, and 3) in the implementation of the initialize function for reading the appropriate values from the configuration file and any other required action for initialization. During initialization and time stepping, the simulation framework will ensure that the new module state is appended and the new module receives the full model state to run its models at each time step.

To add hepcidin, we need to add the required biology to the model by incorporating data that capture hepcidin levels and dynamic processes that correspond to hepcidin biology. The incorporation of data will be handled by expanding the model state; see the center box in Fig. 5. For the purpose of simplicity, hepcidin will be involved in two processes: 1) its own production and 2) macrophage iron handling, an important source of iron in this context. Both of these processes are formalized as submodels within the hepcidin module. Importantly, for the absorption model because of the elementary nature of the macrophage model, either the hepcidin or macrophage module could contain a model in which macrophage data and hepcidin data are read to determine iron absorption. However, if there was an intracellular model for the macrophage within the macrophage module then to expand it to include hepcidin, the macrophage module would necessarily be altered as well. Here, again though, no direct dependencies between the newly added hepcidin module and the macrophage module are introduced in the process.

**Fig. 6.**
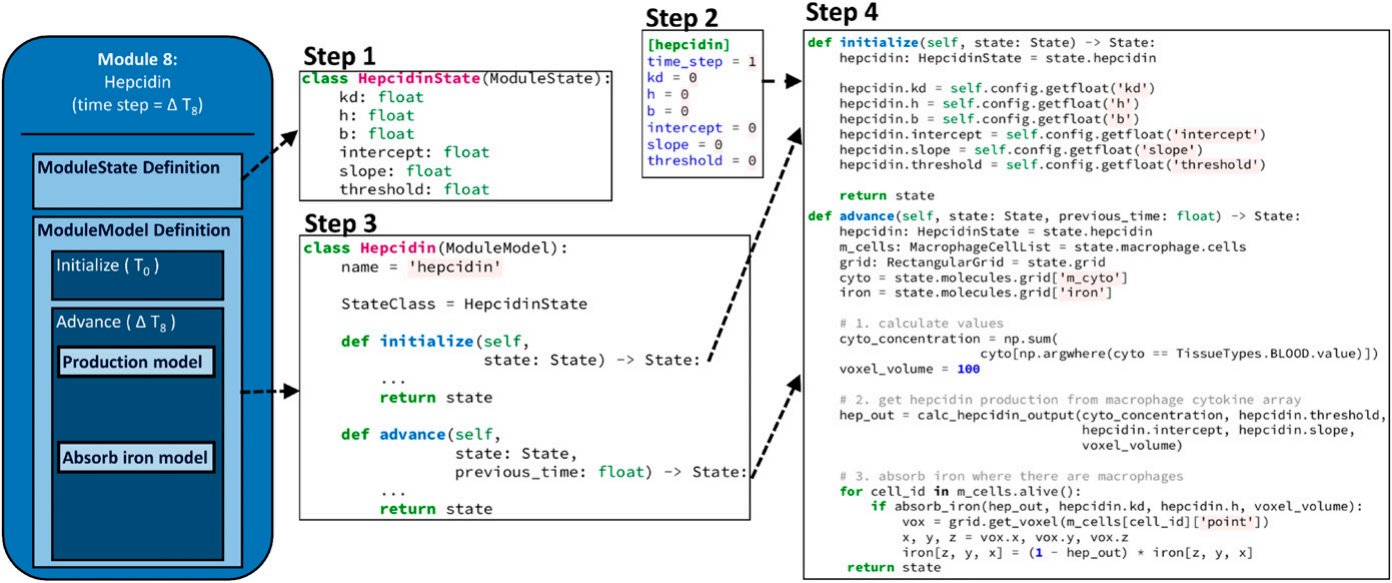
Addition of the Hepcidin module. 1) The hepcidin module must define a subclass of ModuleState, called here “HepcidinState.” Below, all the data related to the ModuleState (six relevant variables) are simply floating-point numbers, but, as noted above, the type of data could also be integers, Boolean, strings, and NumPy arrays of these types. 2) The config.ini file must be altered. The path of the new module file “hepcidin.py” (simulation\modules\hepcidin.py) is added to the modules field under the simulation heading, in the form simulation.modules.hepcidin. Hepcidin, in which Hepcidin is the class extending ModuleModel to be coded in the next step. All the parameters relevant to the hepcidin module are given a value under a new “hepcidin” heading. This section must also include a time_step field, which indicates the period over which the module is to be updated. 3) The third necessary step is to code a subclass of “ModuleModel,” called here “Hepcidin.” Setting values of the “name” and StateClass field are required. 4) The last step is to code the model by implementing initialize() and/or advance() functions. The new hepcidin module will have six parameters, each of which are initialized based on the values provided in the configuration file in the initialize function. Within the “advance” function, all submodels related to the module will be executed. In this example, this includes calculating values, getting a hepcidin number from the macrophage cytokine level, and absorbing iron where biologically indicated.

The newly added logical relationships in the model architecture are indicated in red in Fig. 5. An advantage of the modular architecture is the minimal coding that must be done to make such changes. A detailed description can be found in the *Methods*, including Fig. 6 that provides one potential example implementation.

### Removing a Module.

There are many situations which require the removal of a module, either temporarily or permanently. For instance, in a model of the immune response to a pathogen, the modeler might want to simulate the temporary absence of a cell type, such as the temporary elimination of neutrophils in a patient undergoing chemotherapy treatment. This can be accomplished by setting all requisite parameters in the model equal to zero (e.g., the neutrophil recruitment rate), thereby simulating the module’s absence.

The second method consists of removing a module from the model altogether for instance, because the computational algorithm in the module is to be replaced by a different one. This is conceptually different since we are actually modifying the model by removing or altering the biology that is implemented. This needs to be done with care, just as in a conventional framework. However, the structure described here simplifies the task considerably. Technically, if a module is removed, then its effect on data fields in the global model state is simply removed. In a conventional implementation, this might well break the modified model when it is simulated because of inherent module dependencies. As noted above, to actually remove the computational model from the simulation, one can simply disable the initialize and advance functions of the module containing the model. However, the biological implications of the module’s removal may of course affect the model adversely, if not done carefully.

## Challenges in Model Reimplementation

### Spatial Geometry.

To reproduce the model in ref. 10, the first task was to reproduce the structure of the simulation space and append it to the model state. The model loads the space from a file which was not available, so our simulation space was designed based on a description of the space’s parameters. As such, both spaces are 20 × 40 × 20 (16,000 grid cells) and have approximately the same percentages of tissue types in similar locations (Fig. 4*A*). In our model, 19% of the grid cells make up the air type and 62% comprise nonspecific interstitial tissue cells. For both spaces, four blood vessels run the length of the space, made up of blood cells occupying one grid cell each and comprising ∼6% of the grid cells.

### Parameters.

Determination of parameters also presented a challenge to replication. There are three sources of algorithm description and model parameter values: the paper, the supplemental material, and the code. Because of this and the variation between them, assigning parameter values was not possible in some cases. The molecule-related parameters are all relative values in which the quantity has no intrinsic biological value in isolation, but its relation to other parameters is what determines their importance. It became clear there were multiple configurations used to generate the data as some parameters were reported in multiple places with orders of magnitude difference. Additionally, it was not always clear what time step (hour versus 20-min steps) parameter values were based on. To reconcile this, the parameters were all rescaled to the case in which the cytokine production rate by the epithelium was 100 units per hour or estimated in relation to the others when missing.

### Validation.

Another obstacle to replication came from the lack of raw data from which to do model validation. The paper provided several plots on which we used image digitization software, specifically WebPlotDigitizer (19), to impute the results of simulations. We selected several figures of interest from ref. 10 on which to base our model replication. These included the main figures from the paper as well as a selection from the supplemental material.

## Discussion

As in industry, medical digital twins will play an important role in medicine in the future, not only to treat sick patients better but to prevent them from becoming sick in the first place. The key ingredients for this to happen are, or soon will be, in place: 1) an understanding (still incomplete) of human biology and the homeostatic mechanisms that help us maintain health; 2) ever-improving data that capture the various determinants of our health from genomic sequences to behavioral data; 3) the technologies that allow us to incorporate 1) and 2) into computational models, both mechanistic and data driven; and 4) the increasingly common collaboration between the clinical and the computational sciences needed to create sufficiently credible computational models that have value in the clinic, as tools like the artificial pancreas have shown. What is still missing is the technological infrastructure to combine these ingredients. Unlike the practice in industry, biological and biomedical research is conducted largely by individual investigators around the world collaborating with each other as needed and communicating extensively through conferences, publications, and social media. While this research structure maximizes creativity in research, it requires additional technological infrastructure to “crowdsource” the output of these individual efforts into a coherent whole, for instance, a model that combines all the determinants over the course of a viral infection for an individual patient.

The methodology we present here is an open-source model architecture that satisfies the requirements for distributed model building described here. We discuss some shortcomings of the current version below. It is beyond the scope of this article to discuss the importance of another essential ingredient of distributed digital twin construction, namely, a user-friendly information management system that documents all aspects of the model-building process. This is key for model credibility and integration. We have developed such a system that is structured to match the model structure, making it easy for users to navigate information on experiments, data, analysis pipelines, models, literature, simulation results, and validation. This platform will be published separately.

The expertise needed for using our platform is well within the capabilities of most research groups that engage in computational modeling. The code we provide contains all the components needed to adopt this architecture. And, most importantly, it does not require modelers to substantially change their approach to model building. We took special care to build a tool that has a shallow learning curve and requires few changes in the varied practices of individual modeling groups.

The most important current limitation of the platform we have developed at this time is the fact that all component models in the modules have to be written in Python. Many different languages are in use currently for the construction and simulation of computational models, and it is impractical to limit modelers to a particular one, de facto imposing a standard for anybody wanting to use the platform. A future implementation will allow for the delegation to external programs developed independent of the main simulation and possibly in different languages. We are exploring two possible ways in which this can be done. The first is a containerized system with a standardized system of transferring data focused on simplicity and flexibility. The other is an efficient system which allows programs written in one language to call routines and share data with routines written in another language called “Foreign Function Interfaces” (FFIs). These are supported by a wide variety of languages, including Python, Java, C/C++, Julia, Fortran, Mathematica, etc., and are commonly used in the Python programming community to link high performance numerical functions written in C or Fortran to Python programs (e.g., the NumPy and SciPy libraries). We anticipate that this will be achievable in the current design because the core data structure of NumPy objects are expressible as arrays of C structs, a low-level data type with broad support among FFIs. Libraries can be developed that wrap the low-level C interface into other programming languages, and pointers to the simulation state can be passed directly to external processes through shared memory, retaining the low overhead of the current approach. Assuming that the external process can perform computations directly on this shared state, the data abstraction provided should be capable of executing at near optimal efficiency.

### Integrating Discrete and Continuous Models.

The framework we have developed is not specific to agent-based models or, indeed, discrete models but is generally applicable to all model types, including hybrid models. One problem that needs to be solved for the construction of hybrid models that integrate different modeling frameworks is how to transform data types between models. This problem is the same, whether the model is organized in the modular structure we have developed, in which all modules deposit data in a global model state, or in the conventional way, in which modules pass data to each other directly. There are several possible solutions to converting floating-point numbers to categorical values and vice versa for instance. The choice is up to the modeler. In our use of the framework, we keep data fields that have continuous inputs as floating-point numbers, such as the concentration of a cytokine whose diffusion is governed by a PDE model. If a module with a Boolean model accesses this data field, such as macrophages that secrete this cytokine, then the modeler who built the Boolean model will need to decide how to map zero and one to floating-point numbers, for instance, by using a floating-point threshold that separates the two discrete states.

## Methods

### Modular Architecture.

#### Iteration.

Before any iterations, the solver runs the “initialize” function for each of the specified modules. After initialization is complete, the solver uses an event queue iterator to advance simulation time. The event queue is implemented via Python’s built-in PriorityQueue, which is based on a binary heap algorithm. The event queue is initialized by setting all modules to run at time 0. Once initialized, the solver polls the queue for the next module to advance, advances that module, and adds the module back into the event queue marked by the next time it should be run based on the “time_step” parameter for that module, as specified in the configuration file. Any module with a nonpositive time step is only initialized and not advanced. This process proceeds iteratively until either the queue contains no further events or the current time exceeds the desired simulation length. Note that we do not expect that the queue will run out of events.

A common occurrence is that multiple modules will give the same time step or otherwise have advances which are determined to occur at the same time. In this case, our algorithm updates the modules in the order in which the modules dequeue from the event queue. This order is deterministic but is not necessarily the order of the modules in the configuration file. Future work will explore other options for iteration schemes.

#### Model state.

The simulation’s shared global model state is implemented as an instance of the State class found in state.py. This class serves as a runtime container for simulation configuration/parameters, states of individual modules, and nonmodule-specific parameters such as the clock and the geometry of the spatial grid. The module-specific states are stored in instances of subclasses of the base ModuleState class. The ModuleState class is analogous to a Python “dataclass,” in that its primary function is to store data and only contains code related to initialization and saving said data. When extending a model with a new module, users are expected to create their own subclass of ModuleState. This can be done in a straightforward manner by listing the fields with type annotations. As part of the initialization, the State class instantiates each module’s StateClass field of the ModuleModel implementation, and so, we require that the ModuleState implementation is set to the StateClass field of the ModuleModel implementation. This constructor takes a single parameter, the global state. No default constructor is provided and initialization is expected to be performed in the initialize method of the Module implementation.

#### Module addition.

The conceptual additions to be made to the model to add hepcidin are shown above in Fig. 5. Here, we describe the practical details of implementing a hepcidin (or any other) module. The new module is simply a new Python file named “hepcidin.py” that extends the ModuleState and ModuleModel, as described in general above. The hepcidin model within the module consists of two submodels. Since this addition is for demonstration purposes only, we have chosen a very simple model that relates cytokine levels to hepcidin levels, a simple equation that converts the total macrophage cytokine level into the hepcidin production response, combined with a probabilistic model that governs the effect on a given macrophage. In order to illustrate the simplicity of this process, we describe in detail the template we provide for the code to be added, reproduced in its entirety in the steps in Fig. 6. To emphasize, the code in Fig. 6 is all that is needed in general for the addition of new modules or the replacement of old ones. The only part that needs to be changed for other modules is the inclusion of the actual model to be used instead of the simplified ones we show here.

### Reproducible Code and Dependencies.

Modern code relies on a complex web of interdependent libraries that are in a constant state of development. For example, our simulator has dependencies on 16 publicly available libraries, each of which is either a standard component of Python or available from the Python package index (https://pypi.org). The continual improvement and development of these libraries is a positive feature for the scientific community but presents problems for reproducibility of research. For instance, changes to the API of a package might eliminate, change the name of, or change the behavior of a function. Furthermore, coordinating installation between different operating systems and Python package managers (pip and conda) can lead to troublesome inconsistencies.

We use pipenv (https://pypi.org/project/pipenv/) to provide a consistent “virtual environment” for the simulator. Pipenv creates and manages a copy of the simulator together with specified versions of Python and any package dependencies which are isolated from any other packages present on the user’s machine. The specific versions of Python and package dependencies together, with SHA256 hashes for verification, are listed explicitly in the files Pipfile and Pipfile.lock. One feature of this is that the user need not manage the complexity of obtaining various third-party packages by hand. Another is that by specifying both the version numbers and hashes of libraries it ensures that, to a high probability, the version of the library that is run on a user’s machine is identical to that run on our reference platform.

Furthermore, there are issues of portability between operating systems or even versions and distributions of operating systems and of providing a straightforward installation method for the software. We address these by encapsulating our environment in a Docker container (17). A Docker container provides a common, standardized virtual Linux environment, allowing the same code with the same setup to be run on a wide variety of platforms with minimal setup. Docker is also integrated with the container publishing platform “dockerhub” (https://hub.docker.com/) on which our simulation framework is published.

#### Performance.

A particular concern when designing a modular architecture for performing large-scale simulations is the overhead associated with the software abstraction. All abstractions come at a cost of computation efficiency, and the key to designing an efficient architecture is understanding the source of the inefficiencies and writing low level constructs that avoid them.

Data access latencies increase by many orders of magnitude switching from on-die central processing unit (CPU) cache to higher-level storage media. Roughly speaking, accessing data from CPU cache, random access memory, solid-state drive, and network costs 10, 100, 10^5^, and 10^7^ clock cycles, respectively. In a design which has an excess of data transference between modules, the CPU sits idle most of the time, eliminating any benefit of computational parallelism which might be achieved in a scheme which isolates module data.

Our goal for the modular design was to eliminate as much of the data access overhead as possible. To achieve this, we provide a high-level API that wraps a series of NumPy data structures (11). These data structures are passed directly to the modules by reference, eliminating the need for any data copying or disk/network input/output. This effectively reduces the cost of memory latency for the abstraction to zero.

A natural way to achieve a speed up in computational performance is to parallelize model simulations. Clearly, medical digital twins will be sufficiently complex to require parallel computation. Given the nature of what is being modeled, this is highly nontrivial to do. Efficient parallel algorithms will need to come from the way processes are modeled, separating temporal and spatial scales in ways that allow their computational separation. Parallel execution of model simulation would then be overlaid over our modular framework, which might help parallel computation through a clearer model structure. But by itself, the modular structure does nothing to either aid or hinder parallel computation. In light of this, issues of atomic data access and conflict resolution do not apply to our current implementation.

The stochastic nature of most biomedical models presents other opportunities for distributed computation. Namely, because stochastic models are typically simulated tens or hundreds of times using aggregate statistics to analyze model behavior, these different simulations can be carried out simultaneously on separate computers or processors.

## Data Availability

Our simulator is released as open-source software under the Apache License version 2 (https://www.apache.org/licenses/LICENSE-2.0.txt). It has been packaged for distribution via Python’s standard package manager, “pip,” and is available on the Python Package Index under the name “nlisim” (https://pypi.org/project/nlisim/). A docker container with a complete environment for the simulator is available at https://hub.docker.com/r/nutritionallungimmunity/nlisim. Source code for the simulator is maintained on the public GitHub repository (https://github.com/NutritionalLungImmunity/nlisim). All other study data are included in the article.
